# Supplementary figures and images for: Partial domain adaptation enables cross domain cell type annotation between scRNA-seq and snRNA-seq
Source: PLoS Comput Biol. 2026 May 6;22(5):e1014223. doi: 10.1371/journal.pcbi.1014223 (PMC13170964; doi:10.1371/journal.pcbi.1014223)

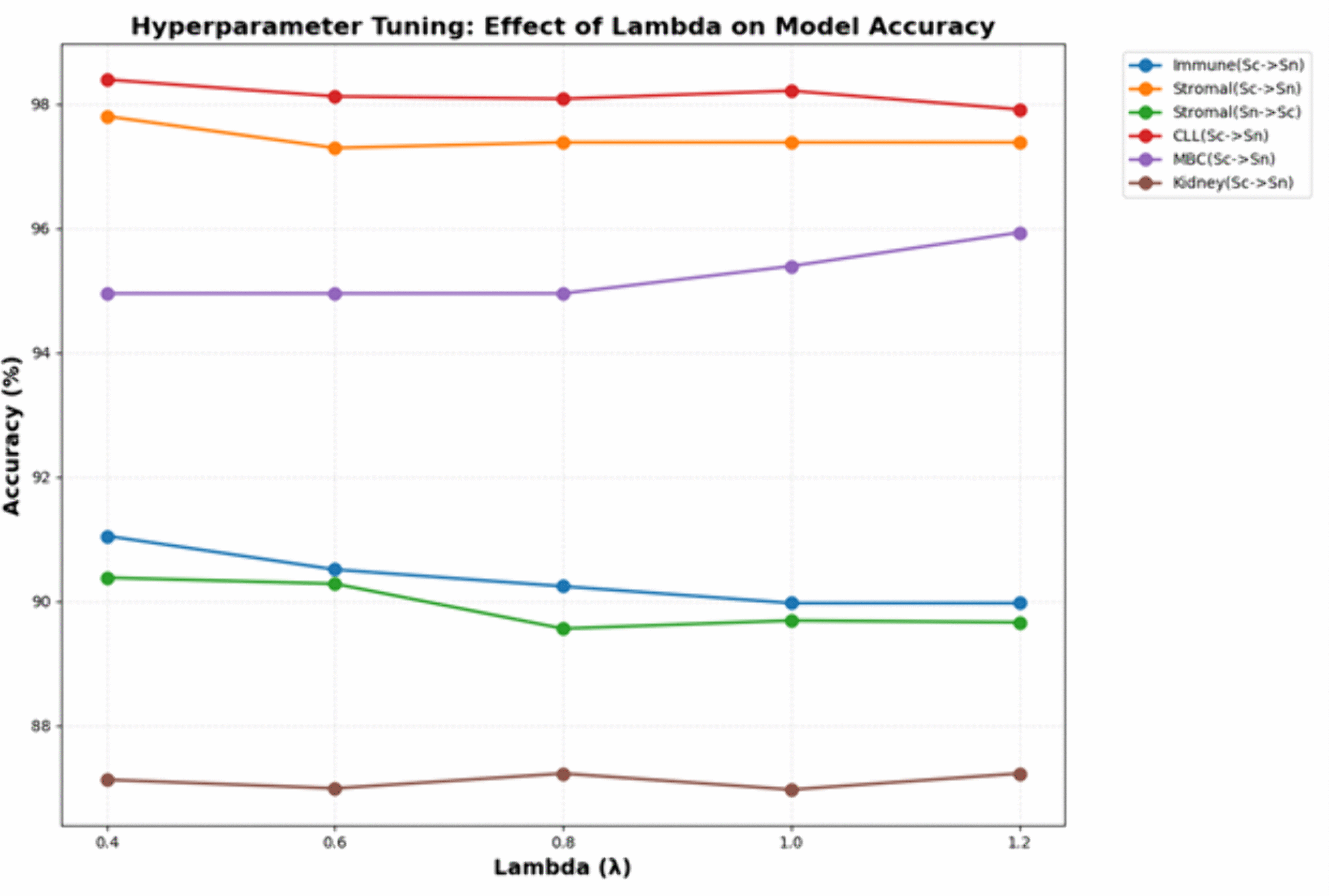

Supplement: S1 Fig — (TIF) [file pcbi.1014223.s001.tif]

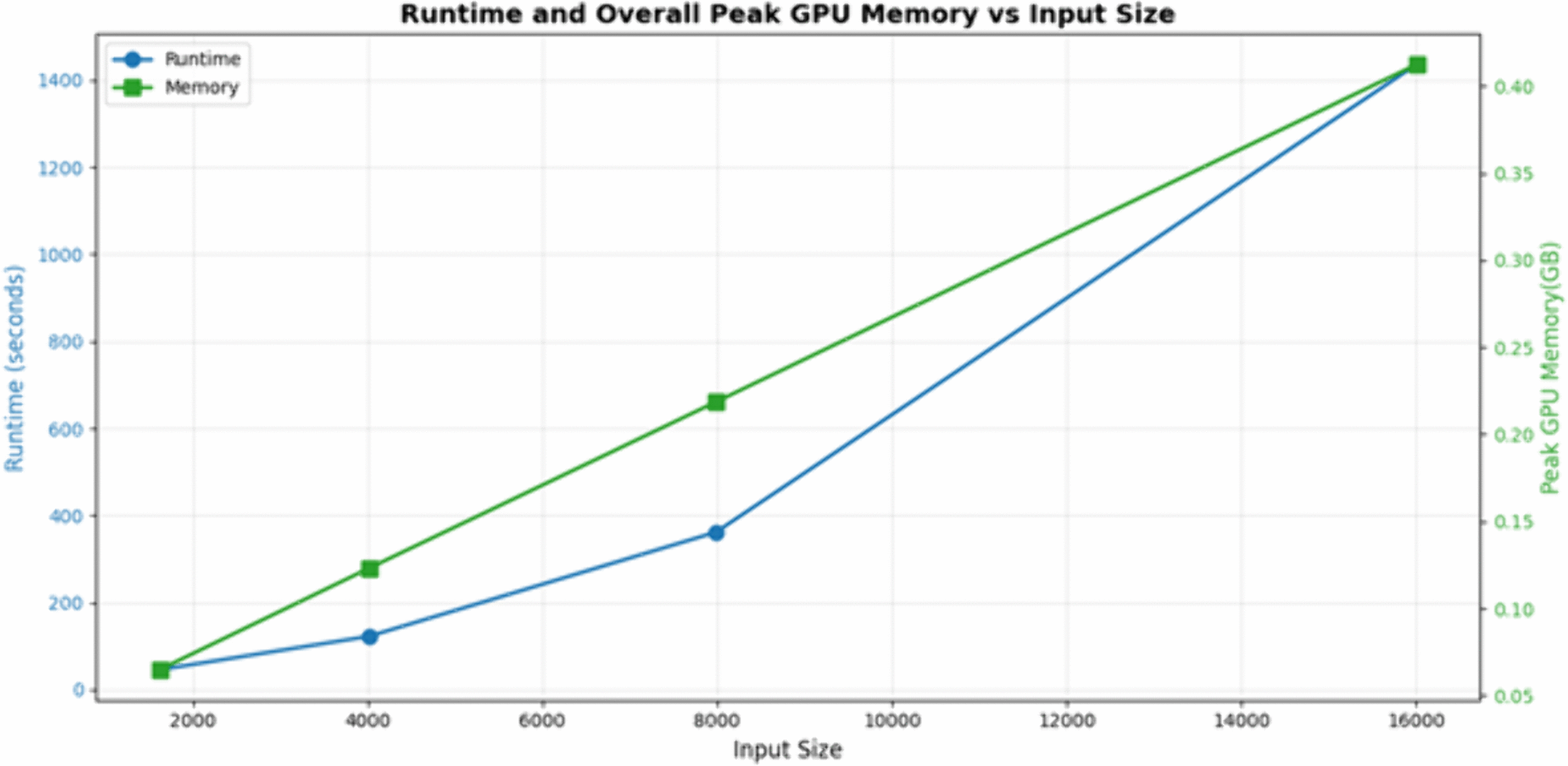

Supplement: S2 Fig — (TIF) [file pcbi.1014223.s002.tif]

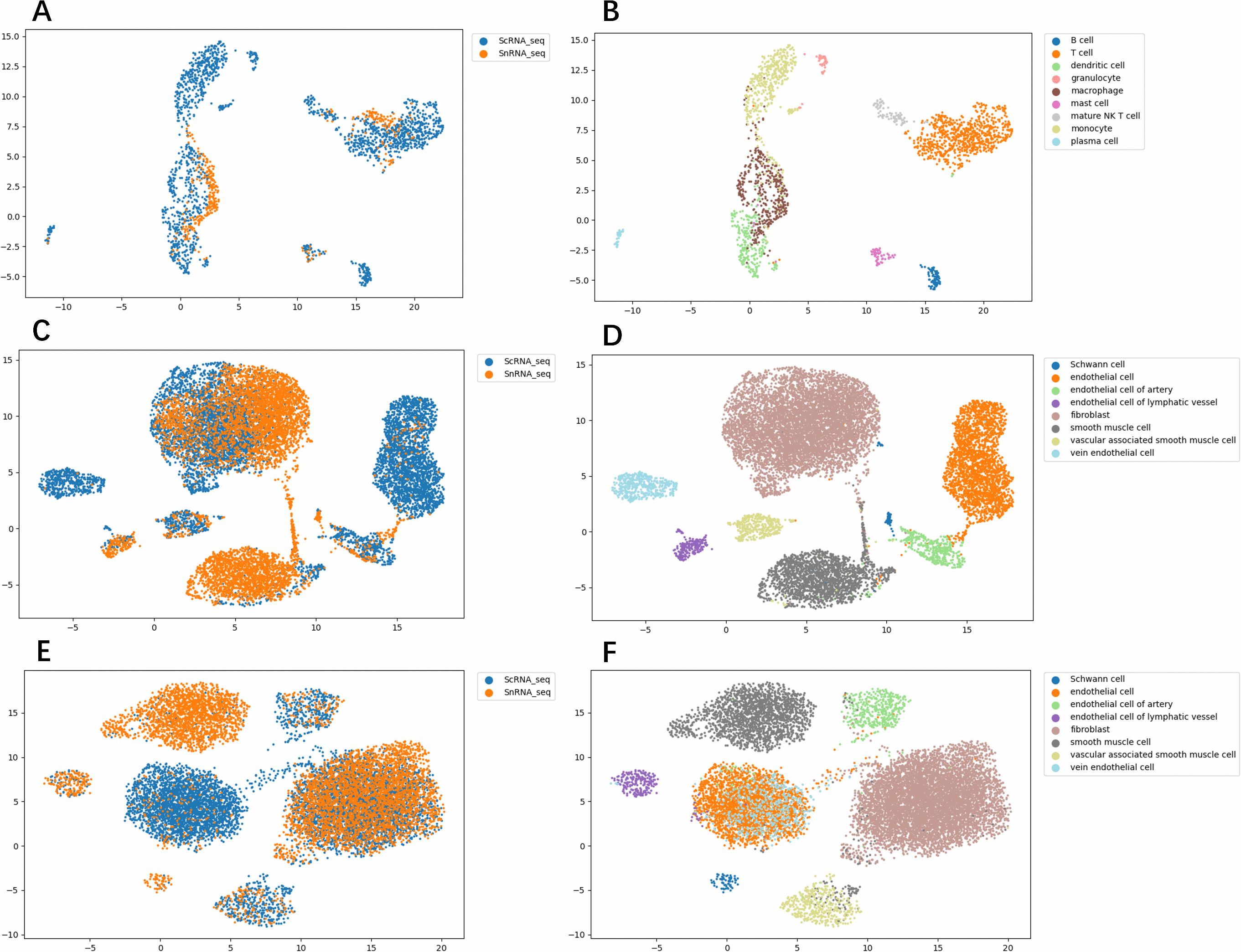

Supplement: S3 Fig — Using UMAP on bladder tissue (A) visualization result on scRNA-seq to snRNA batch representations using immune subset (B) visualization result on scRNA-seq to snRNA cell type representations using immune subset (C) visualization result on scRNA-seq to snRNA batch representations using stromal subset (D) visualization result on scRNA-seq to snRNA cell type representations using stromal subset (E) visualization result on snRNA-seq to scRNA batch representations using stromal subset (F) visualization result on snRNA-seq to scRNA cell type representations using stromal subset. (TIF) [file pcbi.1014223.s003.tif]

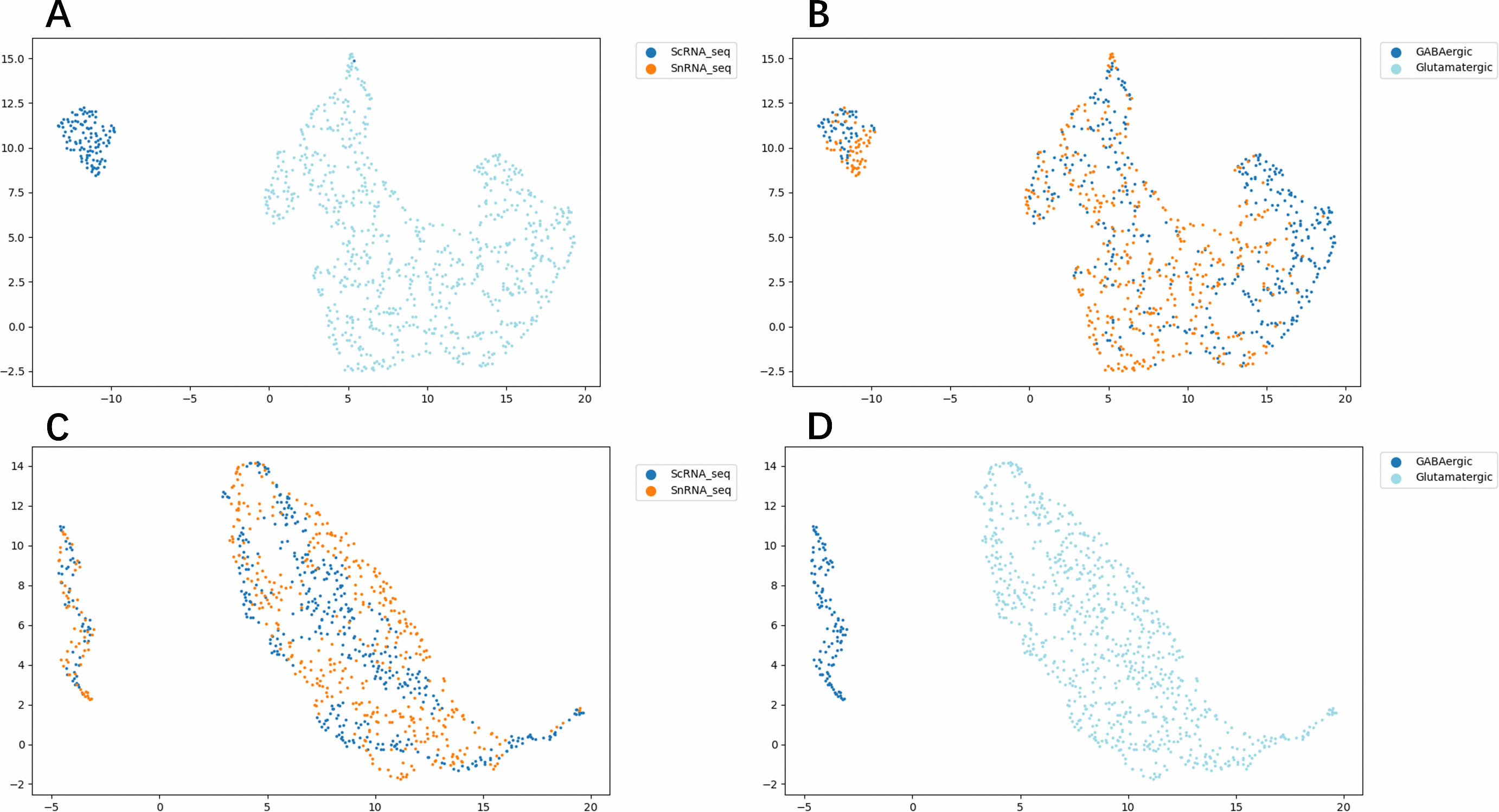

Supplement: S4 Fig — Using UMAP on mouse cortical tissue (A) visualization result on scRNA-seq to snRNA (B) visualization result on scRNA-seq to snRNA (C) visualization result on snRNA-seq to scRNA batch representations (D) visualization result on snRNA-seq to scRNA cell type representations. (TIF) [file pcbi.1014223.s004.tif]

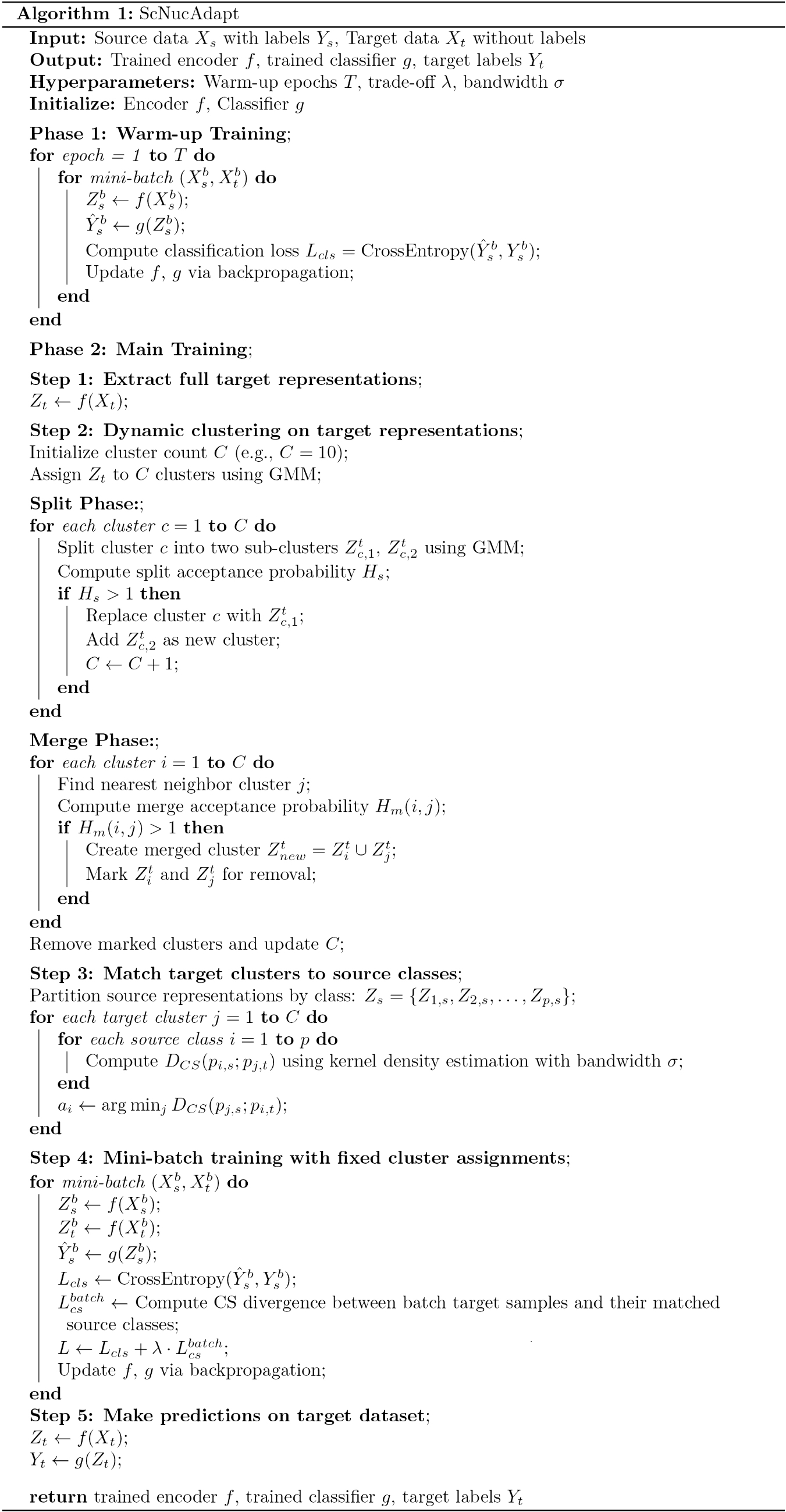

Supplement: S5 Fig — (TIF) [file pcbi.1014223.s005.tif]
