# Supplementary material for: Partial domain adaptation enables cross domain cell type annotation between scRNA-seq and snRNA-seq
Source: PLoS Comput Biol. 2026 May 6;22(5):e1014223. doi: 10.1371/journal.pcbi.1014223 (PMC13170964; doi:10.1371/journal.pcbi.1014223)
Supplement: S1 Table — (DOCX) [file pcbi.1014223.s006.docx]

| ScNucAdapt Hyperparameters | Hiddenlayer Width | Latent dim | early stopping | lambda | lr | batchsize | epoch before GM | K |
| --- | --- | --- | --- | --- | --- | --- | --- | --- |
| immune | 1028 | 1028 | 2 | 0.4 | 0.00005 | 256 | 9 | 9 |
| stromal sc->sn | 32 | 50 | 2 | 0.4 | 0.00001 | 128 | 200 | 9 |
| stromal sn->sc | 64 | 40 | 2 | 0.4 | 0.00001 | 128 | 200 | 9 |
| cll | 32 | 50 | 2 | 0.4 | 0.0001 | 1028 | 200 | 4 |
| mbc | 64 | 50 | 2 | 1 | 0.0001 | 1028 | 2000 | 9 |
| kidney | 128 | 30 | 2 | 0.8 | 0.0001 | 600 | 200 | 10 |
| cortical_sc->sn | 32 | 50 | 2 | 0.6 | 0.0001 | 16 | 200 | 3 |
| cortical_sn->sc | 32 | 50 | 2 | 0.6 | 0.0001 | 16 | 200 | 3 |
