# Supplementary material for: Partial domain adaptation enables cross domain cell type annotation between scRNA-seq and snRNA-seq
Source: PLoS Comput Biol. 2026 May 6;22(5):e1014223. doi: 10.1371/journal.pcbi.1014223 (PMC13170964; doi:10.1371/journal.pcbi.1014223)
Supplement: S2 Table — (DOCX) [file pcbi.1014223.s007.docx]

| Macro-F1 | ScAdapt | ScNucAdapt | ScMap | SingleCellNet |
| --- | --- | --- | --- | --- |
| Immune Sc->Sn | 82.37 | 84.69 | 39.73 | 55.51 |
| Stromal Sc->Sn | 80 | 89.42 | 65.03 | 63.82 |
| Stromal Sn->Sc | 71.61 | 72.47 | 63.72 | 66.96 |
| CLL | 61.34 | 94.47 | 61.03 | 64.05 |
| MBC | 74.89 | 76.16 | 71.17 | 41.23 |
| Kidney | 70.35 | 81.5 | 74.03 | 52.04 |
| Cortical Sc->Sn | 99 | 99.5 | 96.36 | 98.99 |
| Cortical Sn->Sc | 100 | 100 | 97.99 | 100 |
